# Novel adomavirus associated with proliferative skin lesions affecting the dermal denticles of a sand tiger shark (*Carcharias taurus*)

**DOI:** 10.3389/fvets.2024.1470052

**Published:** 2024-10-02

**Authors:** Ashley L. Powell, Alvin C. Camus, John H. Leary, Sarah N. Miller, Cynthia M. Bell, Terry Fei Fan Ng

**Affiliations:** ^1^Department of Pathology, College of Veterinary Medicine, University of Georgia, Athens, GA, United States; ^2^Georgia Aquarium, Atlanta, GA, United States; ^3^Specialty Oral Pathology for Animals, Geneseo, IL, United States

**Keywords:** adomaviridae, genome sequencing, histopathology, denticle, elasmobranch, odontogenic epithelium

## Abstract

A captive sand tiger shark (*Carcharias taurus*) presented with progressive, hard, raised, miliary skin lesions localized to the lateral trunk and peduncle. Histopathologic evaluation of biopsy samples revealed dysplastic proliferation of odontogenic epithelium with the production of collagenous material. Inclusion bodies and viral particles were not observed with light or transmission electron microscopy, respectively. However, using next generation sequencing with Illumina MiSeq and PCR followed by Sanger sequencing, the complete genome of a novel adomavirus, tentatively named sand tiger shark adomavirus (STAdoV), was obtained from the affected tissue. The genome was circular and 18.5 kilobases with bidirectionally transcribed genes, namely EO1, EO2 & 4, EO3, LO4, LO5, LO6, LO7, LO8, and SET. *In situ* hybridization using RNAscope® technology and a STAdoV specific probe localized viral DNA to the nuclei of proliferating epithelial cells. Adomaviruses are an emerging viral group with structural and replicative genes sharing a complex evolutionary history with adenoviruses and small circular DNA tumor viruses including papillomaviruses and polyomaviruses. Adomaviruses are described in a number of fish species in association with both necrotizing and proliferative diseases. BLAST analysis of the viral genome revealed greatest nucleotide identity (71.29%) to guitarfish adomavirus (GAdoV), another elasmobranch virus associated with proliferative (epidermal) skin lesions. Lesions in the index animal persisted for approximately 1 year during which time four conspecifics developed similar proliferations. Ultimately, lesions in all sharks regressed spontaneously without recurrence for 2 years.

## 1 Introduction

“Adomaviridae” is a proposed family of viruses described in teleost and elasmobranch fishes thought to share a complex evolutionary history with adenoviruses and other small circular DNA tumor viruses, including papillomaviruses and polyomaviruses, based on homologies between their replicative strategies and structural components (1). The adomavirus genome contains a gene encoding a replicase protein with a superfamily 3 (SF3) helicase domain, similar to either the papillomavirus EO1 protein or polyomavirus large T antigen, which are proposed to delineate “alpha” and “beta” subgroups within the family. Adomaviruses also encode LO6 and LO8 gene arrays, homologous to adenovirus morphogenetic genes pX and adenain, respectively (1). While adomaviruses are currently considered recombinants of these viral groups, their genetic elements are phylogenetically discrete (1).

The first reported adomavirus was associated with endothelial cell necrosis in Japanese eels (*Anguilla japonica*) in 2011 (2), followed by an outbreak of hemorrhagic gill disease in marbled eels (*Anguilla marmorata*) in 2012 (3, 4). Since then, adomaviruses have been described from proliferative skin lesions in a giant guitarfish (*Rhynchobatus djiddensis*) and smallmouth bass (*Micropterus dolomieu*) (5–7). Most recently, a novel adomavirus was associated with necrohemorrhagic gill disease in American eels (*Anguilla rostrata*) cultured in China (8). A growing list of additional adomavirus sequences and genomes have been identified within genomic sequence data of multiple fish species and other vertebrates (1, 9).

Descriptions of viral diseases in elasmobranchs are limited, despite their popularity in public aquaria and concerns over declining wild populations. In a retrospective study of 1,546 elasmobranch diagnostic cases, 15 (0.97%) were attributed to viral etiologies (10). Early diagnoses of herpesvirus and adenovirus infections were based on histopathologic and electron microscopic findings, namely observation of inclusion bodies and morphologically compatible viral particles, while others were entirely presumptive (10–12). Detailed descriptions of viral genomes are limited to the guitarfish polyomavirus-1 (GPyV) (13) and guitarfish adomavirus (GAdoV) (6). Histopathologic descriptions, electron microscopic findings and limited sequence data on an alloherpesvirus induced branchitis in a tiger shark (*Galeocerdo cuvier*) has also been reported (14).

Described herein are gross and histologic changes associated with unique proliferative skin lesions involving the odontogenic epithelium of dermal denticles in an aquarium exhibited sand tiger shark (*Carcharias taurus*). Following histopathologic evaluation of the lesions, the possibility of a viral etiology was considered. Further investigation utilizing electron microscopy, next generation sequencing, and *in situ* hybridization identified a novel alpha adomavirus that was localized to epithelial cells within the lesion, including cells with ameloblast morphology. A description of the associated adomavirus genome is presented.

## 2 Materials and methods

### 2.1 Case history and clinical evaluation

**Figure 1:**
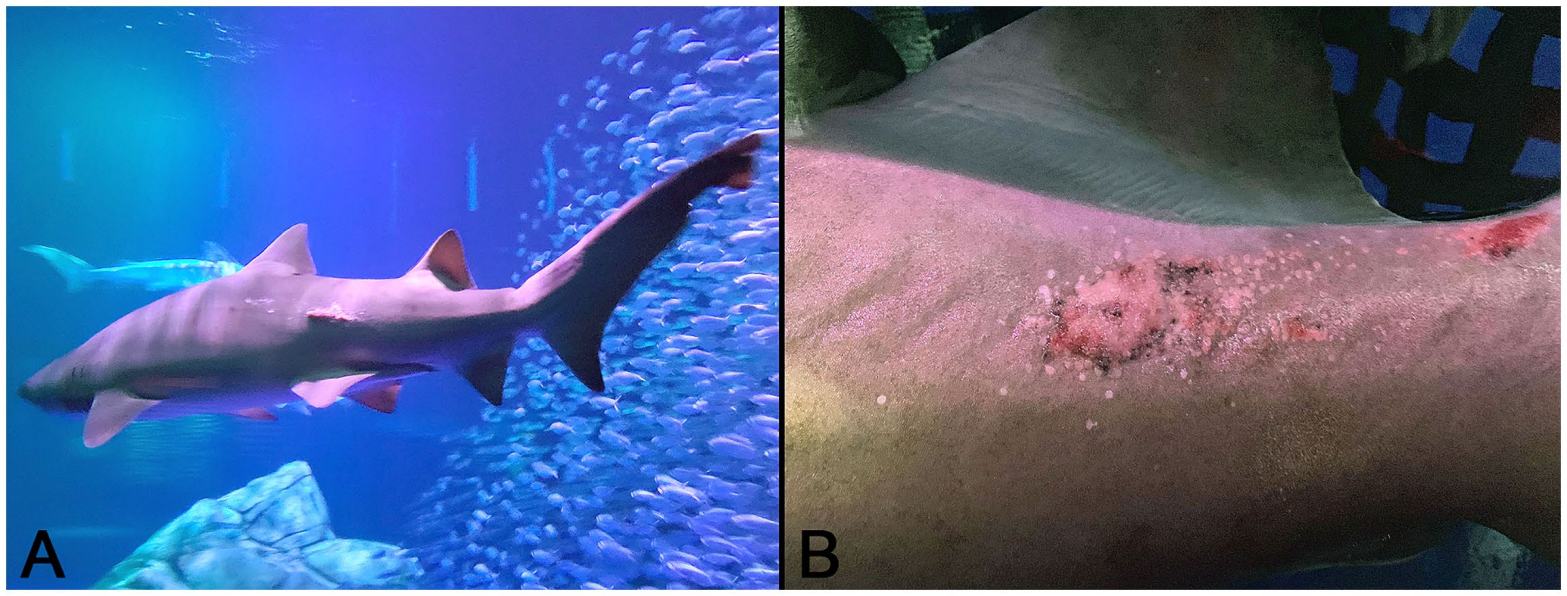
Gross images of the affected sand tiger shark (*Carcharias taurus*): **(A)** Irregular, raised, pink skin lesion on the lateral midline of the caudal trunk; **(B)** Close up image of the coarsely granular proliferative skin lesion composed of numerous, small, hard, coalescing nodules radiating multifocally from the central ulcerated mass.

An estimated 100 kg, wild-collected, adult sand tiger shark (*Carcharias taurus*) maintained for 17 months in an indoor, 1.2 million-gallon, water-recirculating, mixed species, aquarium exhibit developed progressive skin lesions localized to the left lateral trunk and peduncle (Figure 1A). The irregular, miliary lesions were hard, raised, and light gray to pink with small peripheral ulcerations (Figure 1B). Over a period of several months, the skin lesion expanded locally then manifested on the right side. At this time, the shark, which was behaviorally conditioned to enter a stretcher, was manually restrained tank-side and 3% mepivacaine (1.7 mL total) administered by local injection for biopsy sample collection. A full-thickness, 5 mm-diameter skin punch biopsy and two 4–5 mm superficial skin shaves of the lesion were collected from the right lateral peduncle. Separate samples were immediately placed in 10% neutral buffered formalin and electron microscopy fixative containing 2% glutaraldehyde, 2% paraformaldehyde, and 0.2% picric acid in 0.1 M cacodylate-HCl buffer for submission to the Aquatic Pathology Service, Department of Pathology, College of Veterinary Medicine, University of Georgia. Additional samples were frozen at-80°C. Cell culture was not attempted due to lack of elasmobranch cell lines. Following the approximately 10-min biopsy procedure, a 3-week regimen of oral cefpodoxime proxetil (10 mg/kg) and vitamin C (12.5 mg/kg) three times per week was initiated and daily visual observations for signs of abnormal behavior, feeding responses, and infection were made by veterinary staff. Based on the superficial nature and small size of the biopsy samples, as well as potential detrimental effects associated with repeated restraint, analgesic therapy and topical medications were not administered. During this time, the shark continued to feed and behave normally, and the biopsy sites healed uneventfully.

### 2.2 Light and electron microscopy

Skin biopsy samples were decalcified overnight in Kristensen’s solution, bisected and placed cut surface down in tissue processing cassettes. Routine processing by dehydration and clearing in a series of alcohol and xylene solutions, respectively, was followed by embedding in paraffin, sectioning at 5 μm, and staining with hematoxylin and eosin (H&E) and Masson’s trichrome (MT). Comparative evaluation of normal histology and tissues used as controls in immunohistochemical and *in situ* hybridization studies utilized sections of similarly processed sand tiger and silvertip (*Carcharhinus albimarginatus*) shark skin with fully mature and developing dermal denticles. Tissue sections were prepared from paraffin blocks archived at the University of Georgia. For transmission electron microscopy (TEM), fixed tissues were post-fixed in 1% osmium tetroxide, dehydrated in a series of ethanol solutions, stained ‘en bloc’ with 0.5% uranyl acetate, and embedded in Epon-Araldite (Electron Microscopy Sciences). Ultrathin sections were prepared with a Reichert Ultracut S ultramicrotome (Leica), stained with lead citrate, and examined with a JEM-1011 transmission electron microscope (JEOL USA).

### 2.3 Immunohistochemistry (IHC)

Immunohistochemical staining for cytokeratin used a standard indirect protocol. Briefly, paraffin-embedded formalin-fixed (FFPE) tissue sections were deparaffinized and subjected to targeted antigen retrieval (Antigen Retrieval Citra, BioGenex) and background blocking (Power Block™, BioGenex). Primary and secondary antibodies consisted of anti-cytokeratin cocktail (AE1/AE3) anti-mouse monoclonal antibody (Cell Marque) and biotinylated horse anti-mouse IgG (Vector Laboratories), respectively. Color development utilized a horseradish peroxidase (HRP) labeled streptavidin (Biocare Medical) and Betazoid DAB Chromagen kit (Biocare Medical) followed by counterstaining with hematoxylin. For comparative purposes, cytokeratin IHC was performed on a developing denticle in the skin of a silvertip shark.

### 2.4 Next, generation sequencing and bioinformatics

Total DNA was extracted from previously frozen lesioned skin using the DNAEasy Blood and Tissue Kit (Qiagen) as per manufacturer’s instructions. A next generation sequencing (NGS) library was then generated using a Nextera XT DNA Library Preparation Kit (Illumina Inc., USA) and sequenced using Illumina MiSeq with 2× 150-bp paired-end sequencing reagents by GENEWIZ (Azenta Life Sciences). Reads were assembled using the Geneious (Dotmatics; Version Prime 2022.1.1) *de novo* assembly pipeline. Briefly, paired reads were set, trimmed and filtered using default parameters. Trimmed reads were assembled using the Geneious algorithm with the default setting. The contigs were compared to GenBank non-redundant nucleotide database using (BLASTn) and protein (BLASTx) search. Viral contigs were compared and realigned by Geneious assembly for a draft genome to the subsequent analyses below.

### 2.5 Completion of the viral genome

Closing the circular genome was accomplished using conventional and long-distance PCR methods. For the former, NEB OneTaq reagents (M04080L) were used for standard PCR. Annealing temperatures were determined using NEB Tm calculator for each primer pair. Standard PCR reactions included 30 cycles of the following three steps: 95°C for 30 s, variable annealing temperatures for 30 s, and 68°C for 30–60 s. For long-range PCR, LongAmp Taq DNA Polymerase (NEB M0323S) reagents were used as per manufacturer’s protocol to produce the 3,994 bp amplicon used for targeted Sanger sequencing. Cycling conditions utilized 30 cycles of the following three steps: 94°C for 30 s, 60°C for 30 s, and 65°C for 5 min. Primers pairs were designed to flank the putative gap between the 3′ and 5′ ends of the linear sequence generated by NGS assembly (Supplementary Table 1).

### 2.6 Phylogenetic analysis

Helicase core protein sequences from adomavirus, papillomavirus, and polyomavirus representatives were selected based on a previous study (5) and aligned with STAdoV using MAFFT (15). Certain unpublished adomaviruses in GenBank were not included due to uncertainty of their host and sequence status. Maximum likelihood trees were generated using the PhyML (16), where the best amino acid substitution model was analyzed by Smart Model Selection (17). Tree annotation was performed using FigTree.^1^ Mid-point rooting was performed on the adomavirus-only tree as it was constructed without outgroups.

### 2.7 *In situ* hybridization (ISH)

Localization of viral DNA sequences to affected skin utilized *in situ* hybridization (ISH) with RNAscope® technology (Bio-Techne® Advanced Cell Diagnostics™) performed on FFPE tissue (18). A custom designed target probe pool of 20 ZZ probe pairs was used to hybridize within a 978 bp region (Supplementary Table 2) complimentary to positions 11,387–12,364 (5.3%) of the STAdoV genome (V-Ct-AdoV-C1, Advanced Cell Diagnostics™). Briefly, following the RNAscope® 2.5 HD Detection kit (RED) protocol, unstained histologic sections prepared on charged slides were deparaffinized, dried, treated with hydrogen peroxide, rinsed with distilled water, and immersed in target retrieval solution for 15 min at 99°C. Sand tiger shark adomavirus probe or RNAscope® Negative Control Probe (DapB) was added and incubated for 2 h at 40°C. Detection steps followed the Advanced Cell Diagnostics protocol after which slides were counterstained with hematoxylin followed by nuclear bluing (0.02% ammonium hydroxide) and coverslipping with mounting media (Biocare Medical LLC, Concord, CA). A full-thickness section of microscopically normal skin from the same individual was used as an internal negative control.

## 3 Results

### 3.1 Light and electron microscopic findings

**Figure 2:**
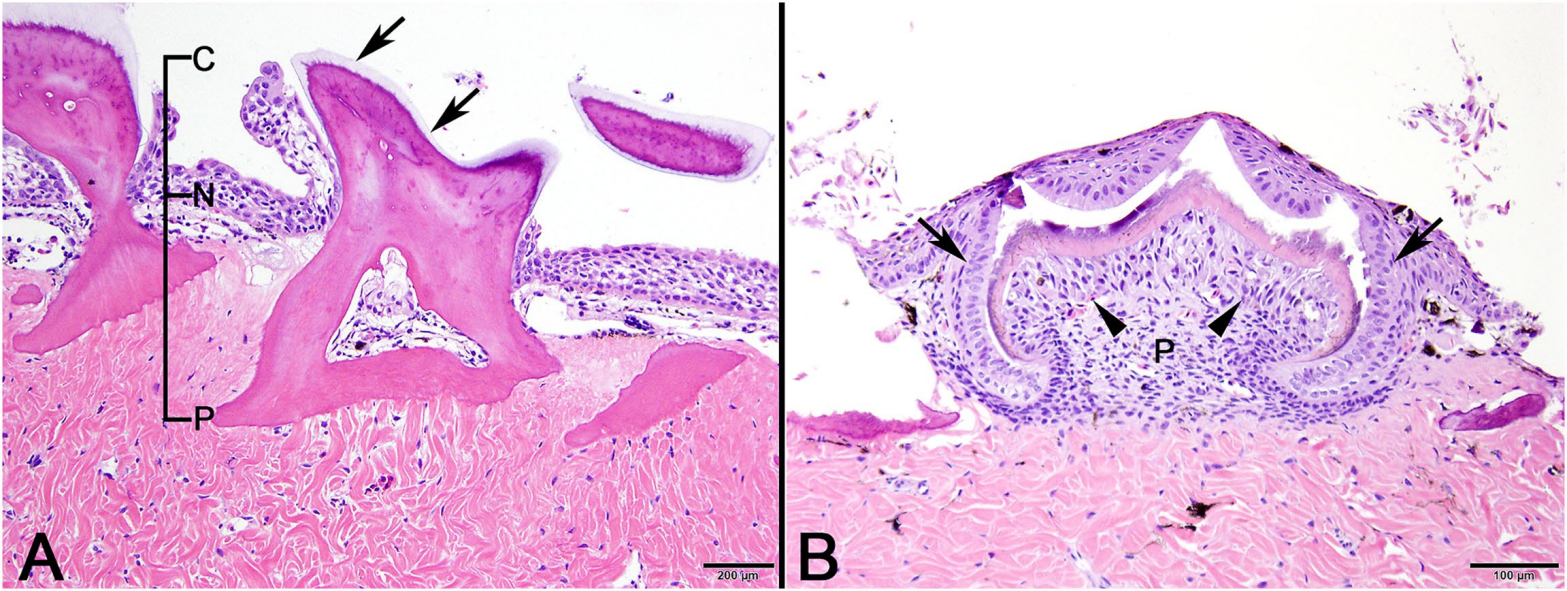
Photomicrographic images of normal sand tiger shark (*Carcharias taurus*) skin with stratified squamous epithelium and dermal denticles. **(A)** Mature denticle from a sand tiger shark composed predominantly of eosinophilic dentine with a broad basal plate (P) embedded in the dermis, narrow neck (N) at the skin surface, and crown (C) with superficial layer of enameloid (arrows). A central pulp cavity is present within the dentine (H&E stain, Bar = 200 μm). **(B)** Developing denticle from a sand tiger shark with thin layers of basophilic enameloid and eosinophilic dentine surrounded by palisading ameloblasts (arrows) and subtended by spindle-shaped odontoblasts (arrowheads). Deep to this is pulp (P) and eosinophilic dense dermal collagen (H&E stain, Bar = 100 μm).

In normal control sand tiger shark skin, mature denticles were composed primarily of acellular dentine surrounding an inner pulp cavity. Denticles were anchored in the dermis by a broad base, tapered as they passed through the epidermis, expanded again above the skin surface and were capped by a thin layer of translucent enameloid (Figure 2A). In developing denticles, dermal papillae were topped peripherally by sequential layers of spindloid odontoblasts, dentine and enameloid with an outer margin of columnar ameloblasts located immediately beneath the surface epithelium (Figure 2B).

**Figure 3:**
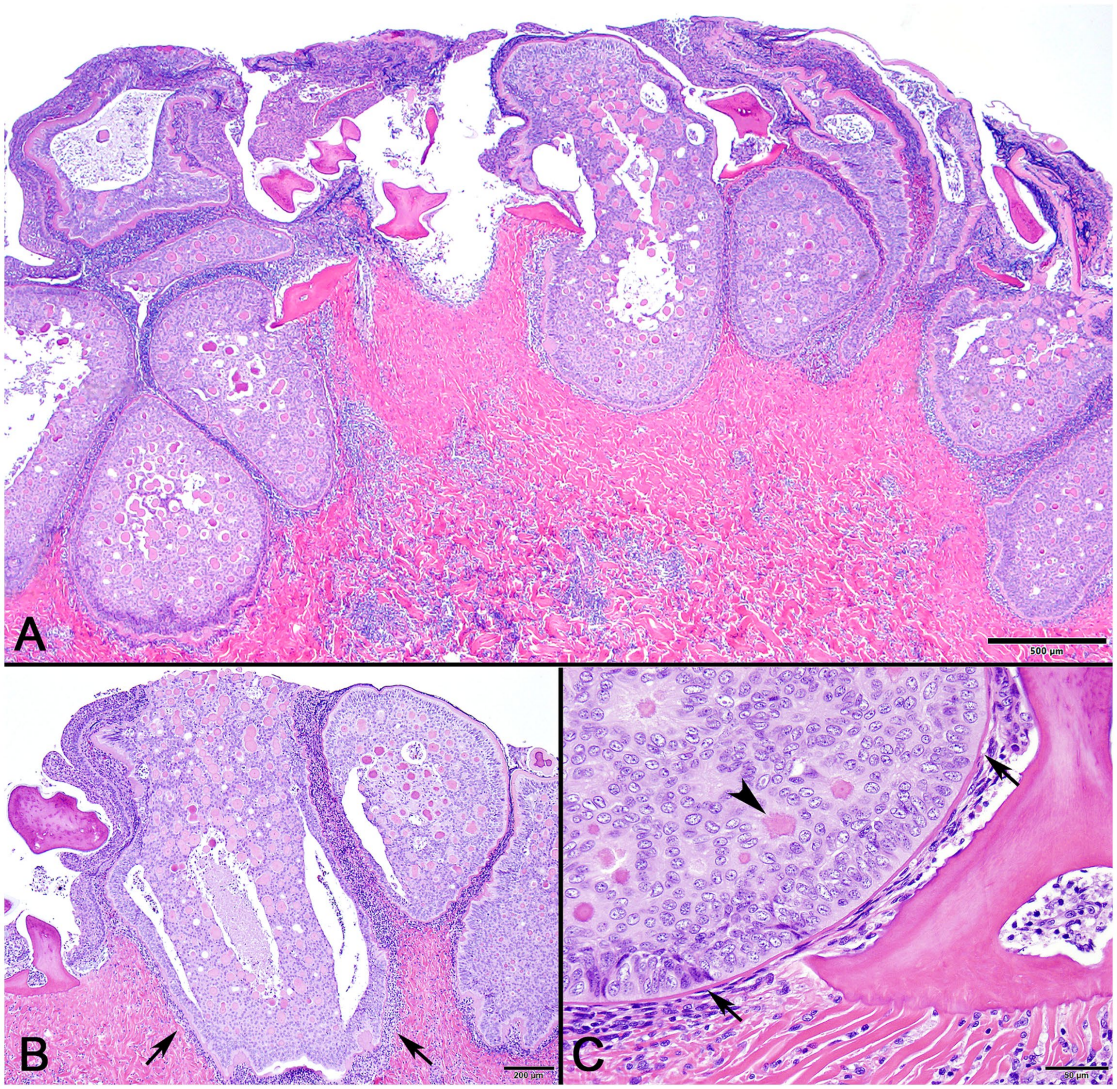
Photomicrographs of lesioned skin from a sand tiger shark (*Carcharias taurus*). **(A)** Lower magnification image of normal surface epithelium and denticle fragments interspersed among multiple large, basement membrane limited structures composed of proliferating odontogenic epithelial cells surrounding eosinophilic islands. (H&E stain, Bar = 500 μm). **(B)** Closer examination illustrates a cavitated space partially filled by necrotic debris within the proliferative lesion. The adjacent dermis is diffusely infiltrated by a dark staining band of lymphocytes (arrows). (H&E stain, Bar = 200 μm). **(C)** Higher magnification image of a proliferative lesion and an adjacent morphologically normal denticle. The lesion is limited by a hyalinized basement membrane (arrows) along which proliferating odontogenic epithelial cells palisade. Internally, follicular-like structures surround eosinophilic islands of hyalinized collagen (arrowhead). (H&E stain, Bar = 50 μm).

**Figure 4:**
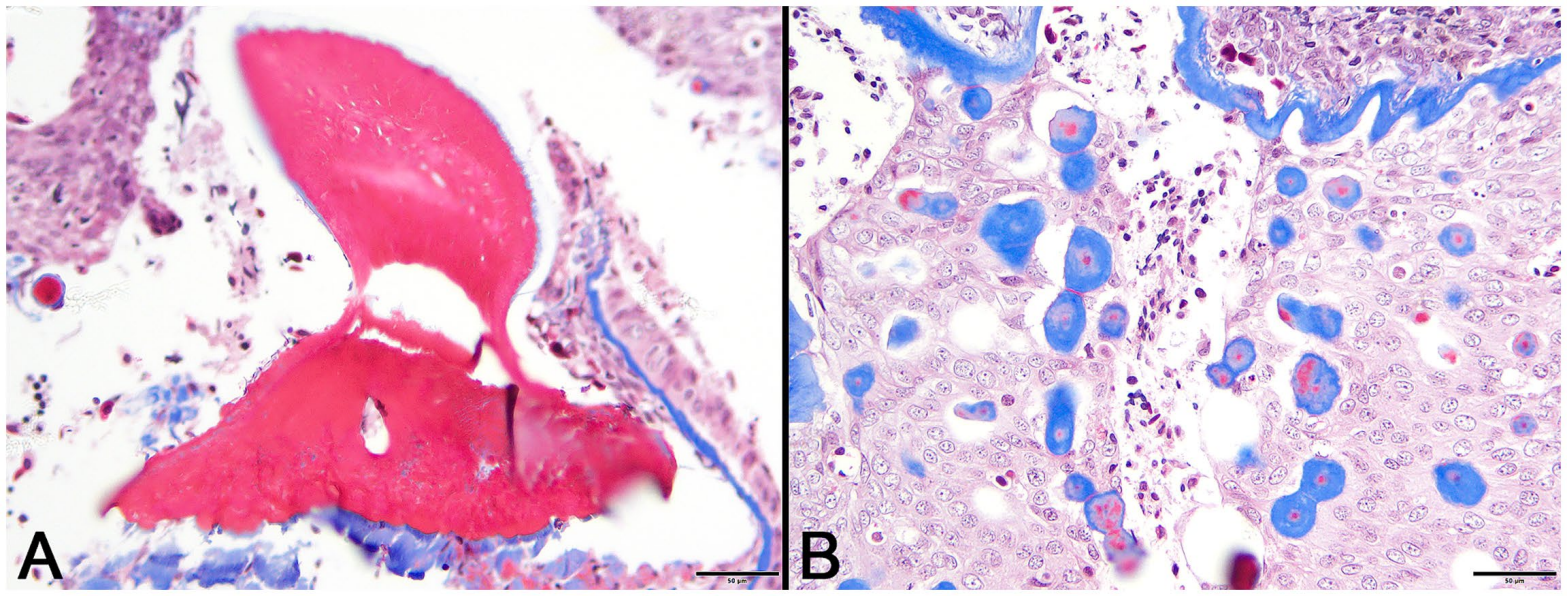
Photomicrographs of Masson’s trichrome sections from lesioned skin. **(A)** Normal denticle stains intensely red. (Bar = 50 μm). **(B)** Blue staining of basement membrane and multifocal islands of material within the lesion are indicative of their collagenous nature. (Bar = 50 μm).

In decalcified sections of lesioned skin, interspersed among morphologically normal dermal denticles, were lobules of proliferative cells corresponding to the grossly visible hard skin nodules (Figures 3A,B). The abnormal structures were limited by a variably thick, hyalinized eosinophilic basement membrane bordered by a zone of palisading tall, thin columnar cells with indistinct cell borders and small amounts of amorphous to finely vesicular pale eosinophilic cytoplasm. Nuclei were ovoid with vesicular chromatin and often a single small nucleolus. Variably shaped polygonal cells with similar cytoplasmic and nuclear features filled central regions of the lobules. These cells were haphazardly arranged or formed follicular structures that encircled small islands of amorphous, vaguely lamellated eosinophilic material that resembled dentine (Figure 3C). With a Masson’s trichrome stain, dentine within normal denticles stained intensely red (Figure 4A). In contrast, thickened basement membranes and the dentine-like material within the proliferative structures stained predominantly blue with only scattered areas of red staining (Figure 4B). This material was interpreted as hyalinized collagen, presumably produced by the proliferative cells but not clearly analogous to either dentine or enamel. The cells had mild nuclear pleomorphism and no mitotic figures were observed. Anisocytosis and anisokaryosis were minimal. Central regions of some affected structures contained cavitated spaces partially filled by necrotic cellular debris (cystic degeneration). Rare lobules of proliferative cells had a small invagination at the base into which streamed elongate mesenchymal cells, resembling pulp mesenchyme. No structures suggestive of inclusion bodies were noted. Dermal collagen immediately adjacent to the affected denticles was infiltrated by small to moderate numbers of lymphocytes (Figure 3B).

**Figure 5:**
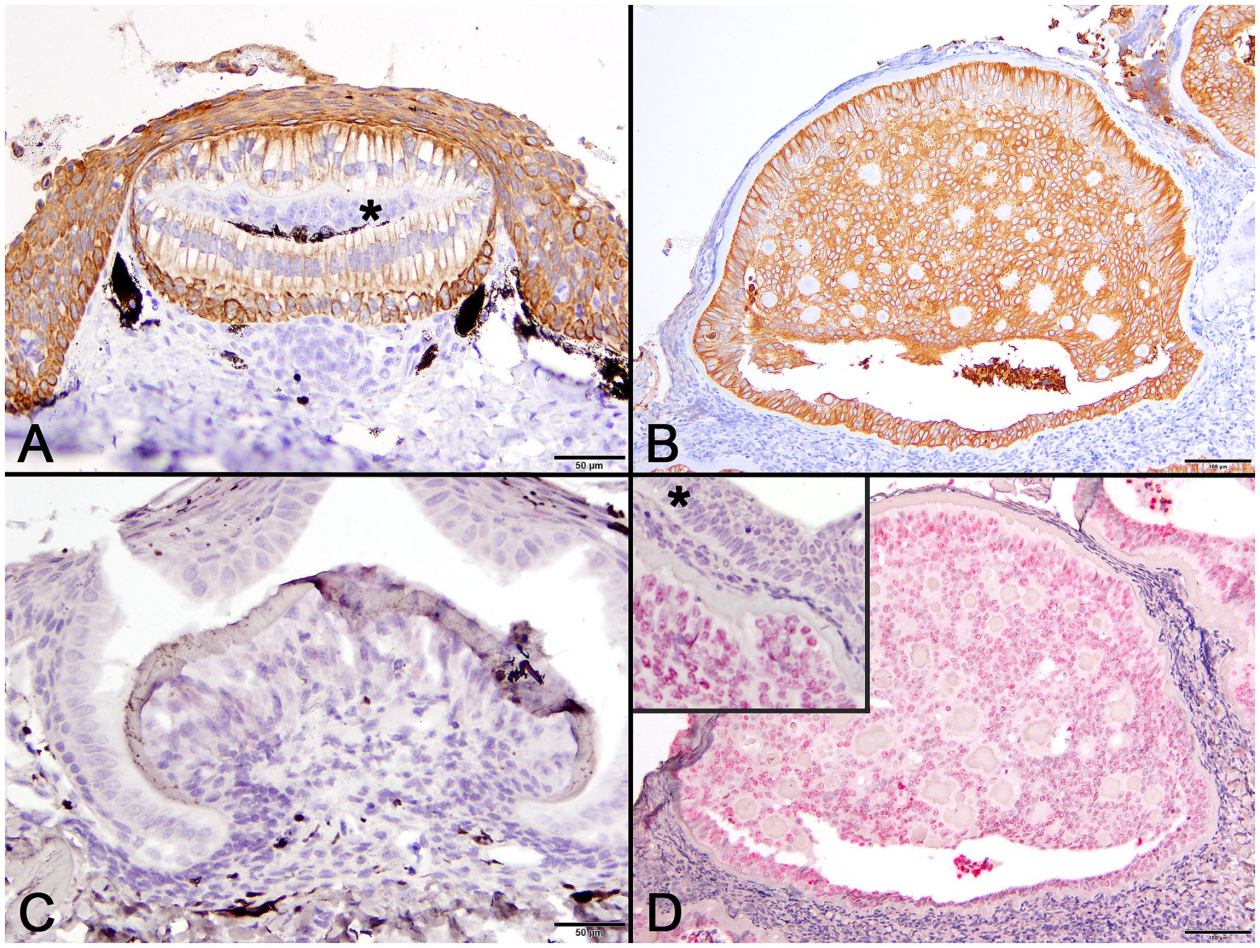
Photomicrographs of normal developing denticles (left column) and proliferative skin lesions (right column). **(A)** Control developing denticle from a silvertip sharp demonstrating positive pancytokeratin (AE1/AE3) cytoplasmic immunoreactivity of palisading ameloblasts and surface epithelium. There is no labeling of centrally located odontoblasts (asterisk) beneath the thin layer of dentine (Bar = 50 μm). **(B)** Positive pancytokeratin (AE1/AE3) cytoplasmic immunoreactivity of odontogenic epithelial cells at the lesion periphery and located centrally surrounding collagenous islands in the affected shark (Bar = 100 μm). **(C)** Normal developing sand tiger shark denticle with no hybridization signal following ISH using an RNAscope® adomavirus probe (Bar =50 μm). **(D)** Strong positive hybridization signal from the nuclei of proliferating odontogenic epithelial cells within lesioned skin of the affected shark following ISH using an RNAscope® adomavirus probe (Bar =100 μm). The higher magnification inset image illustrates a positive hybridization signal within nuclei of odontogenic epithelial cells but not surface epithelial cells (asterisk).

Pancytokeratin (AE1/AE3) IHC was used to identify cells of epithelial origin and served as an internal positive control in normal tissue and the case shark. In a histologic section of a normally developing denticle from a silky shark, cytokeratin IHC positively labeled the cytoplasm of cells comprising the stratified epidermis and the palisading epithelial cells (ameloblasts) forming the enamel organ of the denticle. Odontoblasts, which are neural crest derived ectomesenchymal cells, did not label with the antibody (Figure 5A). In lesions from the affected shark, cytokeratin IHC similarly labeled cells palisading peripherally along the basement membrane, as well as proliferative cells associated with the foci of hyalinized collagen (Figure 5B).

**Figure 6:**
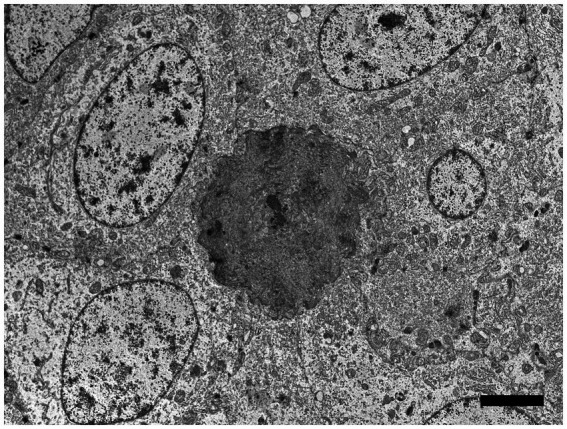
Electron microscopic image of lesioned skin from a sand tiger shark (*Carcharias taurus*) with a cluster of cells surrounding an irregular central island of positively contrasting dentine-like material. Identified as odontogenic epithelial cells, the proliferating cells corresponded to cytokeratin IHC positive cells observed histologically and exhibited a positive intranuclear ISH signal with the RNAscope® probe. The polygonal cells had large central ovoid nuclei and moderate amount of cytoplasm with rough endoplasmic reticulum, scattered mitochondria, and prominent desmosomes. (Bar = 4 μm).

Using the RNAscope® ISH probe, no hybridization signal was detected in an emerging denticle from an unrelated sand tiger shark (Figure 5C). In the case shark, viral DNA was localized by strong positive hybridization signals to the nuclei of proliferating odontogenic epithelial cells within the lesion. There was no labeling of nuclei in the adjacent surface epidermis, odontoblasts or other dermal mesenchymal cells (Figure 5D). Despite the positive ISH results, neither inclusion bodies nor free viral particles were observed in the nuclei or cytoplasm of proliferative cells with TEM (Figure 6).

### 3.2 NGS, genome assembly, and completion

**Figure 7:**
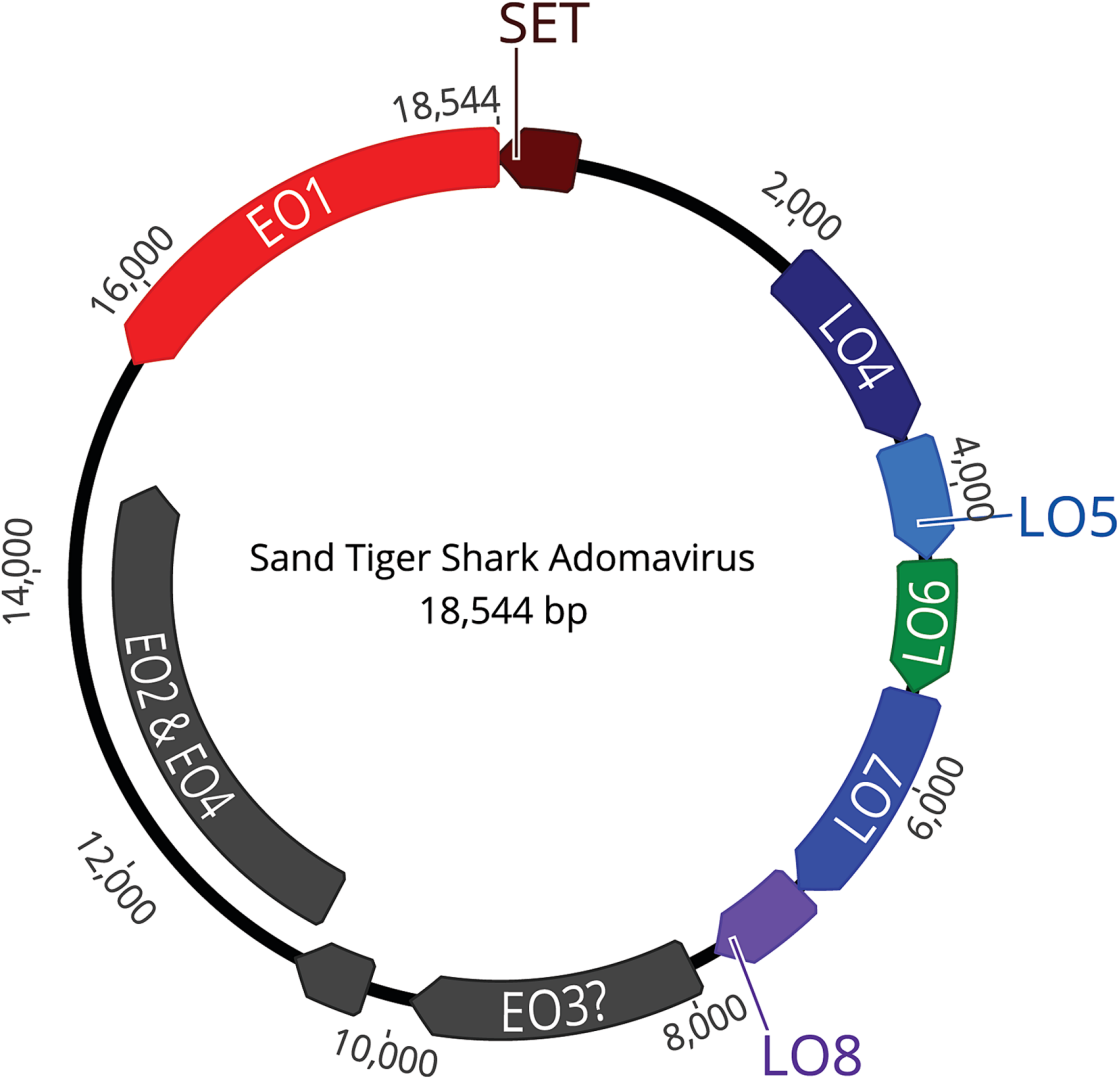
The assembled, approximately 18.5 kb, circular, double stranded DNA genome contains 9 open reading frames (ORFs).

NGS using Illumina MiSeq on extracted skin lesion DNA generated a total of 2,372,727 reads; *de novo* assembly resulted in 368,335 contigs, of which 33,000 were greater than 1,000 bp. The contigs were used to assemble a near-complete adomavirus-like genome, and PCR followed by Sanger sequencing was used to close the circular genome by resolving a 21 bp GC rich sequence region. Final assembly of the circular genome using both NGS and Sanger sequences confirmed a viral genome size of 18,544 bp (Figure 7). NGS coverage spanned the entire genome except for the 21 bp sequence gap near nucleotide position 15,164, which was resolved by Sanger sequencing with 6X coverage. The complete genome shared 71.29% nucleotide identity with the Guitarfish Adomavirus 1 (GAdoV) genome (GenBank MF946548.1) and has been deposited in GenBank under accession number (PQ069217).

### 3.3 Genomic characteristics of the sand tiger shark adomavirus (STAdoV)

The viral proteins of STAdoV were divergent to known viral species but were most related to the adomaviruses (Table 1). The genome encodes two cassettes of bidirectionally transcribed genes, namely EO1, EO2 & 4, EO3, LO4, LO5, LO6, LO7, and LO8, as well as a SET [Su(var)3–9 Enhancer-of-Zeste and Trithorax] homolog (Figure 6). These genes are located on the sense strand, except for EO1 (anti-sense), in a genome organization consistent with those of guitarfish adomavirus and marbled eel adomavirus. Most of the viral proteins are highly divergent, sharing only 38–46% amino acid identity (AI) to the closest adomaviral homologs in GenBank (Table 1). The LO5, LO6, LO7, and LO8 genes showed significant protein homology (Blastx/Blastp) to adomaviral capsid subunits, namely penton, core, hexon, and adenain, correspondingly. While the L04 gene has no significant viral homolog, based on its location, it could potentially code for fiber or cement protein (2). The EO3 gene was inferred to be DNA primase-like, and another gene (EO2 & 4) is named for its location but shares no currently known protein homologs.

**Table 1:** Sand tiger adomavirus protein characteristics and their closest related homologs.

Protein	Length (bp)	Direction	Putative functions	Protein identity to the closest virus	Closest virus in GenBank
LO4	1,440	Forward	Unknown	No significant Blastx hit (currently)	N/A
LO5	825	Forward	Penton	38%	Adomaviruses
LO6	918	Forward	Core	38%	Adomaviruses
LO7	1,569	Forward	Hexon/Major Capsid protein	42%	Adomaviruses
LO8	735	Forward	Adenain	30%	Adomaviruses
EO1	3,024	Reverse	Helicase	39%	Adomaviruses
EO2 & EO4	4,026	Forward	Unknown	No significant Blastx hit (currently)	N/A
EO3	2001	Forward	DNA primase-like	41%	Adomaviruses
SET	558	Reverse	Su(var)3–9, Enhancer-of-zeste, Trithorax (SET) domain superfamily, containing lysine methyltransferases	46%	Adomaviruses

**Figure 8:**
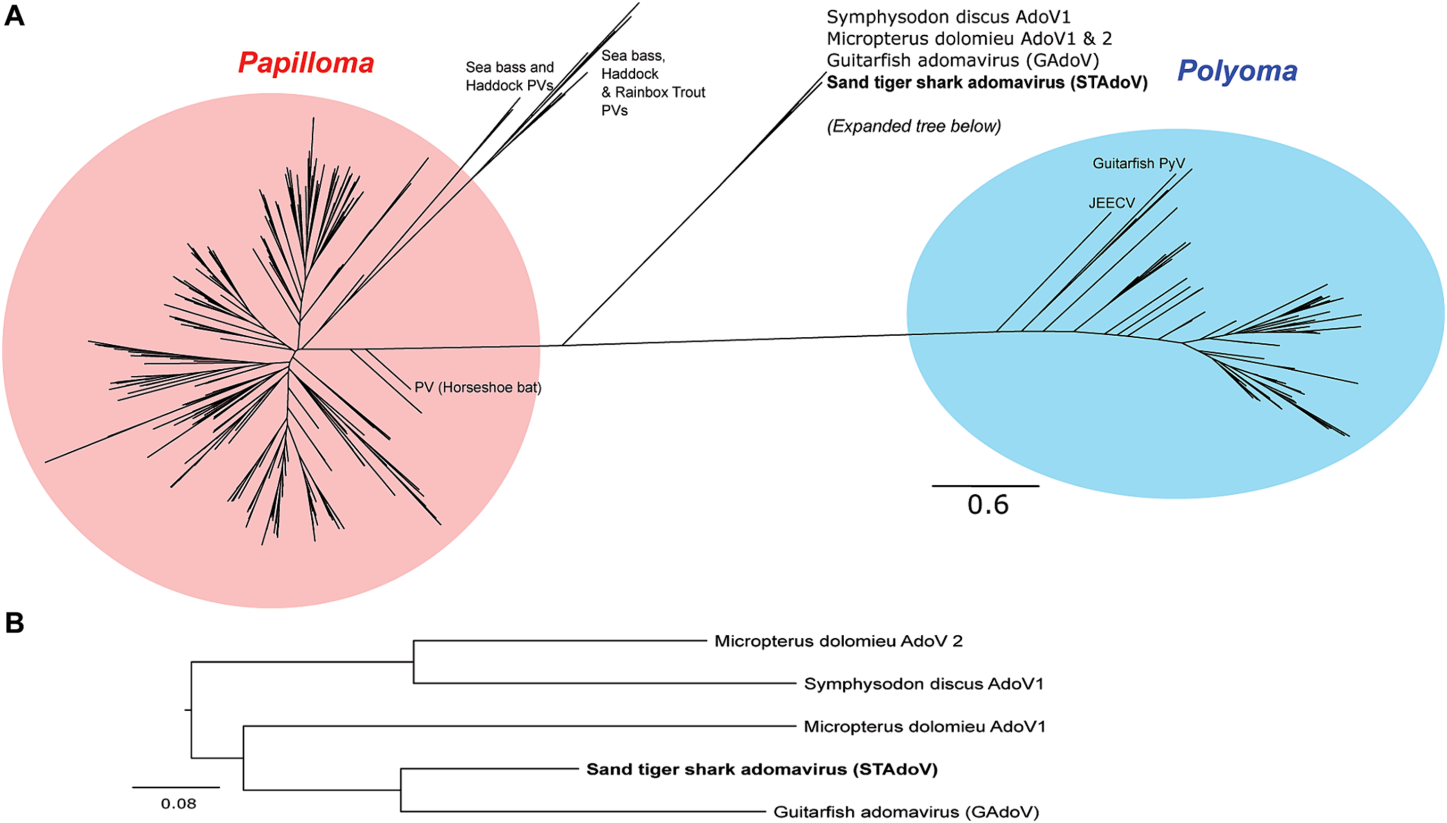
Maximum likelihood trees demonstrating relationships between core helicase proteins of adomaviruses and related taxa. **(A)** Helicase protein phylogeny among adomavirus, papillomavirus, and polyomavirus representatives. **(B)**Helicase protein phylogeny among adomaviruses related to STAdoV. JEECV, Japanese eel endothelial cell-infecting virus; PV, papillomavirus; PyV, polymavirus.

The most conserved protein for adomaviruses is EO1 helicase which is suitable for inter-and intra-viral family phylogenetic analysis (6). Helicase phylogeny of STAdoV showed that it is most closely related to other fish adomaviruses. Consistent with previous observations (6), these fish adomaviruses are distant relatives of polyomaviruses and papillomaviruses (Figure 8A). Considering the genome organization and helicase phylogeny, STAdoV should be recognized as a taxon within the fish adomavirus group. STAdoV could potentially be considered a novel species once criteria are established for declamation by the International Committee on Taxonomy of Viruses (ICTV).

## 4 Discussion

Histopathologic and immunologic examination of the hard, raised skin lesions in the case shark revealed viral associated dysplastic proliferation of cytokeratin-positive epithelial cells, including cells with ameloblast morphology. These lesions represent proliferation of odontogenic epithelium that normally forms dermal denticles in the skin of sharks. Individual lobules within the lesion were limited by a hyalinized basement membrane and these discrete structures likely represent proliferation of the enamel organ epithelium that would have formed a single denticle. Organization of the lesions was reminiscent of ameloblastoma, particularly follicular and dentinoid-associated subtypes as described in humans (19–23). Hyalinization of basement membranes is a common feature of proliferative lesions involving odontogenic epithelium (24). Many small foci of hyalinized collagen were distributed among the proliferating odontogenic epithelium and were presumably mineralized based on hardness of the gross lesions. The precise nature of this material is unclear but suggestive of dentinoid, a poorly understood collagenous material resembling true dentine but having a close association with odontogenic epithelium rather than odontoblasts (25). Hyalinized eosinophilic material within odontogenic epithelial tumors has been variously described as basement membrane, amyloid, glycoprotein, keratin, and enamel matrix (24, 25). In this case, blue staining with Masson’s trichrome supports the collagenous nature of the material. Subepithelial hyalinization and hyalinized collagen deposits may represent unsuccessful attempts by odontogenic epithelium to produce dental matrix in the absence of appropriate induction (24, 26).

Next, generation sequencing and additional PCR yielded the complete, 18.5 kb, circular genome of a novel adomavirus tentatively named sand tiger adomavirus (STAdoV). Using RNAscope® technology, a subsequently developed ISH assay localized viral DNA to the nuclei of proliferating odontogenic epithelial cells within the abnormal denticle-like structures. This is the second adomavirus genome associated with proliferative epithelial lesions in the skin of an elasmobranch and the first described from a sand tiger shark (5, 6).

Similar to infection with the guitarfish adomavirus, lesions in this sand tiger shark were restricted to epithelial cells, although different specific cell types (epidermal versus odontogenic) were involved. The skin of cartilaginous fishes is covered by a stratified squamous epithelium and contains mineralized dermal denticles (placoid scales) that share the same evolutionary origin (odontodes), gene regulatory network, and histologic developmental stages as mammalian teeth, making them serial homologs (27). Functioning to facilitate laminar flow of water and reduce drag, denticles grow to a definitive size, are shed asynchronously, and are then replaced (28). Histologically, denticles are composed of a calcified basal plate embedded in the dermis, tapering neck region, and crown that rises above the surface epithelium (Figure 2A). A central pulp cavity is enclosed by a thick layer of dentine and thin surface margin of enameloid (28). In the developing denticle, a dermal papilla containing mesothelial cells (odontoblasts) is capped by epithelial cells of the stratum germinativum and pushes outward as the base extends into the dermis. Odontoblasts peripherally line the central pulp, produce the dentine matrix and induce overlying epithelial cells (ameloblasts) to deposit the enameloid matrix (27–29) (Figure 2B).

Prior to this investigation, guitarfish adomavirus (GAdoV) represented the only adomavirus described in an elasmobranch species (6). Helicase phylogeny suggested that STAdoV shared a close relationship to a group of piscine adomaviruses including those found in *Micropterus dolomieu* (smallmouth bass)*, Symphysodon discus* (red discus), and giant guitarfish (GAdoV) (Figures 8A,B). This group of piscine adomaviruses have a helicase protein homology that is closer to those of papillomaviruses, thus placing them within the proposed “alpha group” of adomaviruses (1) Among the piscine adomavirus infections for which lesions have been described, members of the “alpha group” appear pathologically more benign and proliferative (5–7). In contrast, the “beta group,” which possess a polyomavirus-like LT helicase and include adomaviruses described from cultured Japanese, marbled, and American eels are highly pathogenic and associated with necrohemorrhagic disease (1–3, 8).

Despite genomic similarities to GAdoV and localization of viral sequences to the nuclei of proliferating odontogenic epithelial cells, neither viral inclusions nor virions were observed for STAdoV histologically or with transmission electron microscopy, respectively. This contrasts with lesions induced by GAdoV, where large numbers of intranuclear inclusion bodies corresponded to arrays of 75 nm icosahedral virus particles (5, 6). We speculate that STAdoV virion production is potentially supported by relatively few cells limiting the chances of observing viral particles in a manner similar to papillomavirus infections (30). A lack of histologic and immunohistochemical evidence of papillomavirus infection has been reported in tumors induced by bovine papillomavirus in feline sarcoids and in human papillomavirus infections where viral DNA was localized to proliferating fibroblasts using *in situ* hybridization (31). It is also conceivable that since the lesions persisted for approximately 6 months prior to sampling, the productive stage of the viral infection had ceased and there were no longer detectable viral particles.

As seen with GAdoV infection in the giant guitarfish, STAdoV-associated skin lesions in the sand tiger shark persisted for many months before regressing spontaneously (5, 6). Histopathologic evidence of regression is presumptively indicated by cavitation with necrotic debris within some lesions and possibly by lymphocytic inflammation in the adjacent dermis (Figure 3). Throughout the course of infection, signs of systemic illness were not observed clinically and residual effects were limited to irregularly shaped areas of depigmented skin. While similar lesions appeared on four conspecifics in the exhibit, they also regressed spontaneously and have not reappeared in over a year indicating the self-limiting infection may produce prolonged immunity. There was no spread to other elasmobranch species in the mixed exhibit, suggesting the virus is species-specific.

Adomaviruses are reported from a variety of fishes (including elasmobranchs), amphibians, and terrestrial and aquatic reptiles with varying clinical significance. Several have been discovered while screening public genomic databanks rather than during investigations of disease, suggesting some may be orphan viruses or nonpathogenic (10, 32). It is speculated that additional adomaviruses will be described as non-biased sequencing methodologies come into more widespread use in routine diagnostics, particularly when applied to the investigation of relatively benign disease processes in uncommonly observed species such as this sand tiger shark. Only a small number of viral diseases are described in elasmobranchs and few reports characterize viral genomes (5, 6, 13, 14). This, however, is changing as molecular diagnostic technologies improve and become more affordable, and interest in elasmobranch conservation and research expands. Cases in which a viral etiology is suspected warrant further attention and investigation, particularly in confined settings conducive to disease transmission.

## Data Availability

The datasets presented in this study can be found in online repositories. The names of the repository/repositories and accession number(s) can be found in the article/Supplementary material.

## Footnotes

^1^ http://tree.bio.ed.ac.uk/software/figtree/
