# Supplementary material for: Novel adomavirus associated with proliferative skin lesions affecting the dermal denticles of a sand tiger shark (Carcharias taurus)
Source: Front Vet Sci. 2024 Oct 2;11:1470052. doi: 10.3389/fvets.2024.1470052 (PMC11480009; doi:10.3389/fvets.2024.1470052)
Supplement: Supplementary file 1 [file Table_1.docx]

**Novel adomavirus associated with proliferative skin lesions affecting the dermal denticles of a sand tiger shark (*Carcharias taurus*)**

Ashley L. Powell^1^, Alvin C. Camus^1*†^, John H. Leary^1^, and Terry Fei Fan Ng^1†^

**Supplementary Table 1**: Primer sequences and functions for PCR and Sanger sequencing of sand tiger shark adomavirus.

| **Primer Name** | **Updated Name** | **Sequence** | **Function** |
| --- | --- | --- | --- |
|  |  |  |  |
| Ad2_395R | 18545R | CTTGACACATTGCGGCAGG | Left End Anchor Amplicon & Sanger Seq |
| Ad2_278F | 18428F | TGCTCTCCTTTTCCACTGCC | Left End Anchor Amplicon & Sanger Seq |
| Ad2_14947F | 14552F | GGTGGTAGACATTGGCTCCC | Right End Anchor Amplicon & Sanger Seq |
| Ad2-15089R | 14649R | CGTCGGCTGATAACAAAGGC | Right End Anchor Amplicon & Sanger Seq |
|  |  |  |  |
| Ad2_804F | 15398F | GTGTATGATGGCCCTGGAGC | Sanger Sequencing Only |
| Ad2_15514F | 15519F | CCTATTCCCATGCCAGACCC | Sanger Sequencing Only |
| Ad2_1227F | 15721F | TTGTGGCTTGGTAAATGCGC | Sanger Sequencing Only |
| Ad2_1430F | 16024F | TGATAGCTCCTGCGTAACCG | Sanger Sequencing Only |
| Ad2_809F | 16578F | TGATACCTGACTTGCTCCCG | Sanger Sequencing Only |
| Ad2_1101F | 16870F | CGGGACCGACACTAATAGGC | Sanger Sequencing Only |
| Ad2_2618F | 17212F | CTGCCCTCTTCCTTGTAGCC | Sanger Sequencing Only |
| Ad2_2821F | 17415F | GTGGAACTAGTGTGTGGGGG | Sanger Sequencing Only |
|  |  |  |  |
| Ad2_626R | 626R | GCTTGTCAGGTACAGAGGGC | Sanger Sequencing Only |
| Ad2_948R | 948R | CGGCCATGTTGTAAAGAGGC | Sanger Sequencing Only |
| Ad2_35R | 16617R | CCTTGCATATTGAAGGCCCG | Sanger Sequencing Only |
| Ad2_125R | 17172R | CATTGTCAGATGTGGGTGCG | Sanger Sequencing Only |
| Ad2_164R | 15745R | CAGTAGCGCATTTACCAAGCC | Sanger Sequencing Only |
| Ad2_204R | 18354R | TTCTCAGGGCAAAGCAGGG | Sanger Sequencing Only |
| Ad2_266R | 16849R | TGTGTCACCTTCATGTGCCC | Sanger Sequencing Only |
| Ad2_331R | 17378R | ACCTGCAACCTCAAAGACCC | Sanger Sequencing Only |
| Ad2_376R | 15957R | GACATAGGTAGCCCACAGCC | Sanger Sequencing Only |
| Ad2_544R | 17592R | TCCTGAAGGTGAAACGAGCC | Sanger Sequencing Only |

**Supplementary Table 2**: Genomic DNA sequence targeted by the RNAscope® in situ hybridization probe pool (positions 11,387–12,364)

ATGGCACACGGCGGCAACGCTGCATCATACTGGTGTCACAGCAGAAGAAATGTGGCAAAAGTATTATTGCAGATGCCTTGCGGACCCTCTGCATGGGACGCCGCATTACGCTAGACCAGAGAGATGGACGTGAGTTCATGATAGGAAGCGCAGCTGAGGGTAACATAGTAATTATAGAGGACCCTAGTGACAAGGCTATTCACTACATGGACCGCACGCTTAGGGCACACCTTGATGGTGACGCAGTCCCTATCAATATTAAAAACCAACCCATCACCCAGCGGCAATACCCACCAGTAATTATAACCACTAATGCGCCCCTAGCAGCTGAATGCCTGCAGAGCAGAGCCACATGCTTTACGTTCACAAAGCGTATGGGAGACATATTTGGGGGACGCCATGTTGAGCGCATACCTCCATCTGATATCGCATGTATGATTGCAAAATATACAATCATGCCAATGTGTAATGCAATATACGAGTTCAACGATCCTGTACATGCATGGGGCCCACTGTGCTGTAGAGGCGACAAGGCCGATGGGCATGATCCATTCTGTCGGTGGAGTCGCTATCTGCACAGGATTAGTAAAAATATATCACCACGGGAGCACACTATGAACCTGCGCCTTCCAACAGGTATCATGGGAATATGCAAACTATACTATTATGAGCGGATGAAGCGAAAGAACTGTGGCCTCCTGTTAACGGTAGATGCAATACGTGCCGCTACAGACTATATCACACACATGAAAATCTGTGATATACCCCGCGGCGATGATACAAGTGATGCAAAACTTGACGTCAAAAAGTTTTGTGATGGCCTGTTGGGCGCTATGTCATATGTTAACTACAAGCTTTCCCCTGATGGTGAAATGACCTTCATAACAGTGGCACAACCGCACATGTGGGAGGCCTGTAGAAATGGGCTCACACACACTGAGTACTGTGCTCTGCCTGTAGCCACAGCTAAATCAATGA
